# Evaluation of segmentation accuracy and the improvement of time effectiveness using deep learning-based segmentation in ^177^Lu-DOTATATE dosimetry

**DOI:** 10.1186/s40658-026-00897-x

**Published:** 2026-05-24

**Authors:** Tetsu Nakaichi, Yuhei Shimizu, Satoshi Nakamura, Yasunori Shuto, Yusaku Kasai, Atsushi Shishido, Kouji Kunito, Hiroki Nakayama, Mihiro Takemori, Masashi Kawamura, Takahito Chiba, Hiroyuki Okamoto, Tairo Kashihara, Hiroshi Igaki, Kimiteru Ito

**Affiliations:** 1 Section of Radiation Safety and Quality Assurance, National Cancer Center Hospital, 5-1-1 Tsukiji, Chuo-ku, Tokyo, 104-0045 Japan; 2 Division of Research and Development for Boron neutron capture therapy, National Cancer Center Exploratory Oncology Research & Clinical Trial Center, 5-1-1 Tsukiji, Chuo-ku, Tokyo, 104-0045 Japan; 3 Department of Radiological Technology, National Cancer Center Hospital, 5-1-1 Tsukiji, Chuo-ku, Tokyo, 104-0045 Japan; 4 Medical Physics Laboratory, Division of Health Science, Graduate School of Medicine, Osaka University, Yamadaoka 1-7, Suita city, 565- 0871 Osaka Japan; 5 Department of Comprehensive Oncology, Nagasaki University Graduate School of Biomedical Sciences, 5-1-1 Tsukiji, Chuo-ku, Tokyo, 104-0045 Japan; 6 Euro MediTech Co., Ltd, 2-20-4, Higashigotanda, Shinagawa-ku, Tokyo, 141-0022 Japan; 7 Department of Radiology, Hyogo Cancer Center, 13-70 Kitaouji-cho, Akashi City, Hyogo, 673-8588 Japan; 8 Department of Radiation Oncology, National Cancer Center Hospital, 5- 1-1 Tsukiji, Chuo-ku, Tokyo, 104-0045 Japan; 9 Department of Radiological Science, Graduate School of Human Health Science, Tokyo Metropolitan University, 7-2-10 Higashi-ogu, Arakawa-ku, Tokyo, 116-8551 Japan; 10 Department of Diagnostic Radiology, National Cancer Center Hospital, 5- 1-1 Tsukiji, Chuo-ku, Tokyo, 104-0045 Japan

**Keywords:** ^177^Lu-DOTATATE, Theranostics, Artificial intelligence, Dosimetry, Medical physicist

## Abstract

**Background:**

The efficacy of deep learning-based artificial intelligence segmentation (AI-seg) in ^177^Lu-DOTATATE dosimetry remains underexplored. This study evaluates AI-seg’s contouring accuracy, dosimetric reliability, and time efficiency.

**Methods:**

We analyzed 23 patients treated with ^177^Lu-DOTATATE. Four medical physicists (MPs) and four radiological technologists (RTs) manually delineated liver (including lesions), spleen, and kidneys on CT images. The most experienced MP modified AI-seg outputs to establish a reference contour, confirmed by a board-certified physician. Both the manual and AI-seg contours were checked against this reference using the Dice similarity coefficient (DSC), Hausdorff distance (HD), and mean distance to agreement (MDA). Maximum, mean, and minimum absorbed doses were calculated from a single time-point image using SurePlan™ MRT (MIM Software Inc.) to assess dosimetric implications. The total time required for dosimetry—including image reconstruction, segmentation, and absorbed dose calculation—was compared between the manual and reference methods.

**Results:**

Only one liver delineation by an MP required correction. Median (IQR) DSCs for liver, spleen, and kidneys were 0.955 (0.950–0.961), 0.929 (0.898–0.944), and 0.929 (0.918–0.942) for manual, 0.988 (0.946–0.998), 0.965 (0.858–0.995), and 0.994 (0.985–0.999) for AI-seg, respectively. All organs met the acceptable DSC threshold ($\:\ge\:$0.8). Median HD in liver exceeded 10.0 mm for both methods, with AI-seg exceeding 30.0 mm in a few cases (liver: 3/23; spleen: 2/23) due to hepatomegaly. The median MDA in manual and AI-seg was below 2.0 mm. Median mean absorbed doses in the reference was 4.03 (1.69–5.81) Gy for liver, 1.55 (1.30–2.50) Gy for spleen, and 2.03 (1.45–2.44) Gy for kidneys. Only the kidneys absorbed dose from AI-seg differed significantly, showing a 1.4% increase. Dosimetry using the reference method took significantly less time than the manual approach (47.0 [30.0–58.0] min vs. 54.3 [49.5–67.0] min, *p* = 0.014).

**Conclusions:**

AI-seg with minor adjustment enables faster yet accurate absorbed dose estimation in ^177^Lu-DOTATATE. Despite segmentation challenges in hepatomegaly cases, MPs and RTs demonstrated competent contouring performance, supporting collaborative dosimetry workflows with physician oversight.

**Supplementary Information:**

The online version contains supplementary material available at 10.1186/s40658-026-00897-x.

## Background

^177^Lutetium-[DOTA^0^, Tyr^3^] octreotate (^177^Lu-DOTATATE) radionuclide therapy is a treatment option for somatostatin receptor-positive neuroendocrine tumors (NET). It is a highly tumor-specific internal radiation therapy that targets somatostatin receptors overexpressed on tumor cells. It utilizes the cell-killing effect of β-rays, enabling individualized treatment through imaging using γ-rays simultaneously emitted from the ^177^Lu. In a randomized phase Ⅲ trial (NETTER-1) for investigating efficacy in advanced midgut NET, ^177^Lu-DOTATATE plus long-acting octreotide showed a clinically and statistically significant improvement in progression-free survival compared to high-dose long-acting octreotide [1, 2]. Additionally, a phase II trial for evaluating the safety and efficacy of dosimetry-based individualized ^177^Lu-DOTATATE therapy that increases the total number of treatment cycles based on estimated renal biological effective dose showed the potential to further improve treatment outcome, with no grade 3–4 renal toxicity [3].

In order to achieve safe and effective individualized treatment (i.e., adjustment of injected activity and adding additional cycles), it is necessary to manage the administered activity based on accurate absorbed dose calculation for dose-limiting organs [4]. The Medical Internal Radiation Dose (MIRD) schema provides a framework to calculate quantitative radiation absorbed dose and has been used for radiation protection, risk assessment, and treatment planning in nuclear medicine diagnosis or treatment [5]. The traditional framework, calculated using S values obtained from a phantom model of a standard human body, can provide the mean absorbed dose of a standard person. However, it is difficult to evaluate the absorbed dose for individual patients accurately. On the other hand, MIRD pamphlet 17 provides the schema to calculate voxel-level absorbed dose using single photon emission computed tomography combined with computed tomography (SPECT/CT) images, thus enabling more accurate individual absorbed dose calculation and evaluating absorbed dose distribution using spatial dosimetric parameters such as dose-volume histogram (DVH) [5].

In external beam radiation therapy, absorbed dose parameters based on DVH and mean organ absorbed dose are essential indicators for predicting treatment efficacy against tumors and preventing adverse events for organs-at-risk (OARs) [6, 7]. In nuclear medicine radionuclide therapy, radiation’s energy and dose rate differ from those used in external radiotherapy, and the DVH parameters for predicting the dose-response of tumors and normal organs may differ [8]. Furthermore, pharmacokinetics must also be considered for achieving an accurate individual absorbed dose calculation. Therefore, treatment based on three-dimensional dosimetry would be expected to become an indispensable technique in the future development of nuclear medicine radionuclide therapy.

In order to calculate each organ’s absorbed dose using SPECT/CT images, contour delineation of normal tissues must be performed. Generally, these processes are performed manually by a physician with specialized skills and experience [9–11]. However, manual contour delineation significantly increases a physician’s workload [12–14]. To reduce the physician’s workload, it is necessary to effectively use human resources, such as other medical staff with sufficient anatomical knowledge (e.g., medical physicists and radiological technologists). The involvement of medical physicists in dosimetry processes, including contouring, is actively supported in Council Directive declarations (2013/59/Euratom) [15].

In recent years, research on artificial intelligence (AI)-based segmentation using deep learning has attracted attention in the field of radiation therapy, and it has been reported that the accuracy is comparable to that of manual contouring by physicians [16–18]. However, in radionuclide therapy, such as ^177^Lu-DOTATATE for advanced NETs, contouring accuracy, dosimetric accuracy, and time efficacy need to be investigated for applying the automatic segmentation [9–11].

The objective of this study was to investigate the contouring accuracy using automatic segmentation based on deep learning for organs at risk such as the whole liver (including lesions), spleen, and kidneys, and to evaluate its dosimetric impact on dosimetry in ^177^Lu-DOTATATE. Additionally, we compared the required time for delineations in OARs between manual contouring and modifying the AI contour by medical physicists and radiological technologists to determine whether the use of AI segmentation could improve time efficiency in a practical scenario.

## Materials and methods

### General

This retrospective study was approved by the institutional ethics committee of the National Cancer Center Hospital, Tokyo, Japan (approved number: 2023 − 333). A total of 23 patients who received four cycles of ^177^Lu-DOTATATE, at 7.4 GBq per cycle, at 4-week intervals from November 2021 to February 2023 were enrolled in this study. Patient characteristics are summarized in Table 1. Patients were admitted to the hospital after administration of ^177^Lu-DOTATATE, and a SPECT/CT examination was performed after meeting the criteria for discharge in Japan ($\:18\:\mu\:SV/h\ge\:$ 1 cm equivalent dose rate at 1 m from the patient’s body surface). The mean ± standard deviation of the examination time after ^177^Lu-DOTATATE administration was 34.53 ± 24.47 h (median: 24.49 h). No serious renal or hematologic adverse events of Grade 3 or higher associated with the treatment were observed in this cohort.

### Data acquisition

Examinations were performed using the Symbia T6 or Symbia T16 SPECT/CT systems (Siemens Healthineers, Erlangen, Germany). The dual-head gamma camera in the Symbia T6 or T16 is equipped with a medium energy low penetration collimator consisting of a 3/8-inch NaI crystal and 59 photomultipliers.

CT data were used primarily for attenuation corrections and contouring of normal organs. For CT data acquisition, the tube voltage was 130 kVp, and automatic exposure control (Care Dose4D) was used for tube current modulation. CT data was reconstructed with a 700 mm field of view, and a slice thickness of 1.5 mm. Rotation time was 0.6 s.

SPECT data were collected using step-and-shoot mode with a clockwise rotation of 12 min/180° (radius of rotation: auto proximity) with 36 views. The image acquisition matrix size was 128 × 128 (zoom factor: 1.00), and scan range covered from the neck to pelvis. Image acquisition energy centers (widths) of 208 keV (20%) and 113 keV (20%) were used. In the triple-energy window method for scatter correction, the center value (window width) of the energy window was set to 176 keV (11.4%) on the low energy side and 238 keV (8.4%) on the high energy side.

### Image reconstruction and absorbed dose calculation

Image reconstruction was performed using SPECTRA Quant™ installed on MIM version 7.2.7 (MIM Software Inc., Cleveland, OH), utilizing the transferred SPECT projection data and CT images. SPECT reconstruction was performed using an ordered subset expectation maximization algorithm (iteration; 3, subset; 3) with bilinear CT-based attenuation correction and the triple-energy window scatter correction. The full width at half maximum of the post-processing Gaussian filter was 4 mm.

Voxel-level absorbed dose calculation was performed based on MIRD Pamphlet 17 using SurePlan™ MRT (MIM Software Inc., Cleveland, OH) [5]. The voxel S-value convolution kernels are sourced from a publicly available database of kernels simulated in this manner [19]. In the absorbed dose calculation process, cross-calibration factor and CT value-to-electron density conversion table were obtained in each SPECT/CT device. The cross-calibration factors for converting from count per second (cps) to Bq were obtained from a cylindrical water phantom filled with ^177^Lu solution. The cross-calibration factors were 9.64 cps/MBq for Symbia T6 and 9.92 cps/MBq for Symbia T16. The CT value-to-electron density conversion tables, which can transform the Hounsfield unit to mass, were obtained using Gammex Model 467 Tissue Characterization Phantom. (Gammex Inc., Middleton, USA). SPECT imaging can only be performed once at our facility; hence, the calculation of the time-integrated activity curve adopted the Hänscheid approach, a single time point method [20]. This method simplifies the calculation of the integral term of the normalized uptake function involved in calculating self-absorbed doses without estimating the effective half-life in each patient. When the measurement time-point is between 75% and 250% of the effective half-life assumed in the Hänscheid approach, the time integral of the exponential equation can be estimated with an accuracy of 10% compared to the actual integral. Image acquisition should be done 72–96 h after ^177^Lu-DOTATATE administration.

### Comparison of contouring accuracy

Three contouring methods were applied to the CT images of 23 patients after the first cycle of ^177^Lu-DOTATATE to evaluate the efficacy of AI segmentations in reflecting the clinical scenario. In this study, the organs contoured were the whole liver (including lesions), spleen, and right and left kidneys. Kidney contouring includes renal cortex and renal medulla, but excludes the renal pelvis and cold areas such as blood vessels and cysts for all segmentation methods. In the liver, metastatic tumors, intrahepatic bile ducts and arteriovenous vessels were included in the contour because they were not visible on non-contrast CT. The three methods are described as follows.

*Manual contouring*: Eight observers from the nuclear medicine or radiation oncology department participated in the manual contouring process, including four medical physicists and four radiological technologists. These observers were divided into four groups according to clinical experience, with two individuals in each group. For each patient, one observer was randomly selected from each experience-based group to perform organ segmentation. Consequently, each patient’s organs were segmented by four individuals with different levels of clinical experience. Manual contouring was performed using a 2D circular brush (MIM), and the decision to use slice-to-slice interpolation was left to the operator.

*AI segmentation*: AI segmentations were performed on the liver, spleen, and right and left kidneys using Contour Protégé AI installed on SurePlan™ MRT. Contour Protégé AI utilizes neural network models based on the U-net architecture covering 37 types of normal organs [21]. AI segmentation was not intended to segment metastatic tumors in the liver. Additionally, metastatic tumors in the liver could not be excluded from the liver in our patient cohort because of the large number of metastatic tumors in the liver and the complexity of identification using only non-contrast CT.

*Reference contouring*: The AI segmentation was modified by the most experienced medical physicist in nuclear medicine and radiation oncology. A board-certified radiation oncologist reviewed this modified AI segmentation; minor modifications were made to create reference contours if necessary.

The Dice similarity coefficient (DSC) was introduced to evaluate the spatial correspondence of manual contour, AI segmentation, and reference contour. The DSC between two structures (*A* and *B*) is calculated using the following equation, allowing for quantitative spatial agreement evaluation.

$$\:\mathrm{D}\mathrm{S}\mathrm{C}\left(A,B\right)=\frac{2\left|A\cap\:B\right|}{\left|A\right|+\left|B\right|}$$

In this study, the tolerance of DSC was set to 0.800 or higher, according to the previous study [22]. The DSC is commonly used to evaluate the spatial coincidence between two structures. However, quantifying the distance relationship between structures and local segmentation errors is difficult [23]. Therefore, we calculated the Hausdorff distance (HD) and the mean distance to agreement (MDA) between pairs of the three delineation methods to analyze the distance relationship between two structures. HD was calculated using the following.

$$\:\mathrm{H}\mathrm{D}\left(A,B\right)=\mathrm{m}\mathrm{a}\mathrm{x}\left(\mathrm{h}\left(A,B\right),\:\mathrm{h}\left(B,A\right)\right)$$

$$\:\mathrm{h}\left(A,B\right)={\mathrm{m}\mathrm{a}\mathrm{x}}_{a\in\:A}{\mathrm{m}\mathrm{i}\mathrm{n}}_{b\in\:B}\lVerta-b\lVert$$

Where *a* and *b* denote arbitrary points located on the perimeters of structures *A* and *B*, respectively. The quantity $\:\lVerta-b\lVert$ refers to the Euclidean distance between point *a* and point *b*. The MDA concept is similar to the HD and can be obtained by replacing the maximum operation with the mean operation. When the outlines of two structures are entirely consistent, MDA and HD tend to approach zero. The tolerance of HD and MDA was ≤ 3.0 mm according to a previous study [22].

### Comparison of absorbed dose

The mean absorbed dose (D_mean_), maximum absorbed dose (D_max_), and minimum absorbed dose (D_min_) in the OARs, including the whole liver, spleen, and right and left kidneys, were calculated using manual contouring and AI segmentation, and compared to those obtained with the reference contour.

### Comparison of required time

To evaluate the effect of AI segmentation in reducing the time required for delineations, we first compared the time required for manual delineation of individual normal organs, such as the whole liver (including lesions), spleen, and right and left kidneys, with that for the reference contour created with the correction after AI segmentation.

Additionally, the total time required to perform dosimetry of OARs, including the whole liver, spleen, and kidneys, was noted. The total time required included the time spent on SPECT reconstruction, contouring, and absorbed dose calculation. We compared the total time required for manual contouring to that for creating reference contours using AI segmentation with manual contour correction. The time spent in AI segmentation refers to the processing time on the central processing unit (CPU, Intel, Xeon Silver 4210, 2.2 GHz).

### Statistics

Manual contouring and AI segmentation were compared using the Wilcoxon signed rank test to assess contouring accuracy, including metrics such as DSC, HD, and MDA. Additionally, this test was used to compare the dosimetric parameters (e.g., D_mean_, D_max_, and D_min_) of manual contouring and AI segmentation with those of the reference contour. Using the Wilcoxon signed rank test, we conducted an analysis to examine differences in the time required for adopting manual contouring versus creating the reference contour using AI segmentation correction for each normal organ. The same test was also used to compare the total time required between the manual and reference contours for all normal organs in a practical clinical scenario. We conducted comparisons using nonparametric tests owing to a limited sample size. Statistical significance was set at *p* < 0.05. Data are presented as median (IQR, 1st quartile-3rd quartile). All statistical analyses were performed using EZR version 1.61 [24].

## Results

**Fig. 1:**
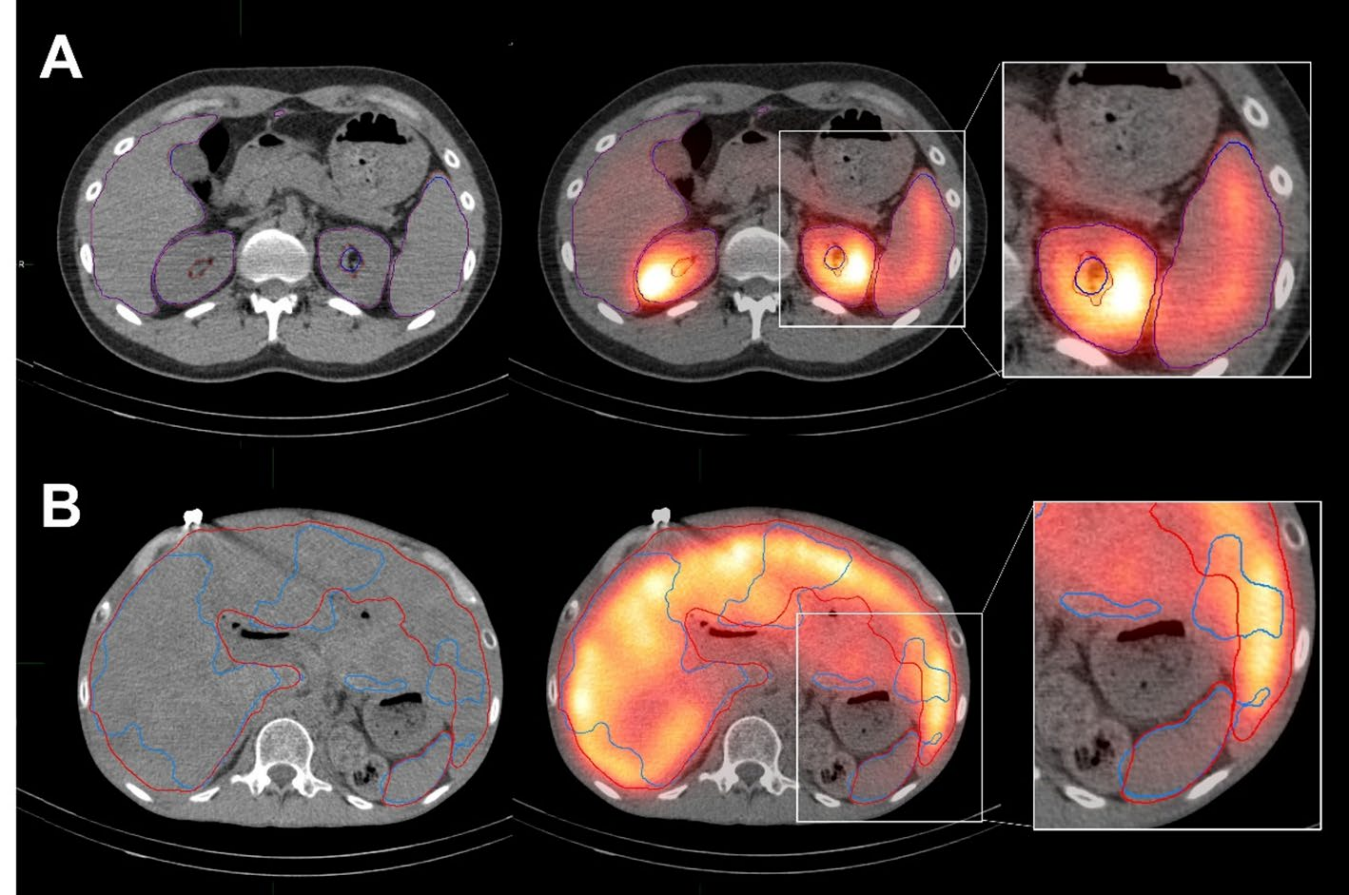
The top images (**A**) were the excellent case, where segmentation of the whole liver (including lesions), spleen, and right and left kidneys was comparatively successful, and the bottom images (**B**) were an example of an incorrect segmentation, where segmentation of the liver and spleen failed. Red lines indicate the reference contour, and blue lines indicate AI segmentation. In the bottom images, the AI segmentation of the spleen (thick blue line) deviates considerably from the reference contour (thick red line). AI; artificial intelligence

Figure 1 shows images from an excellent case, where segmentation of the whole liver (including lesions), spleen, and left and right kidneys was successful, and a wrong case, where segmentation of the whole liver and spleen failed. In the reference contour, only one of 23 liver cases required correction by a certified radiation oncologist. The time required for this case included the time spent on the correction by the certified radiation oncologist. Of the 23 patients, 6 had no spleen because of surgery; hence, 17 patients with a spleen were included in the analysis.

### Contouring accuracy

**Fig. 2:**
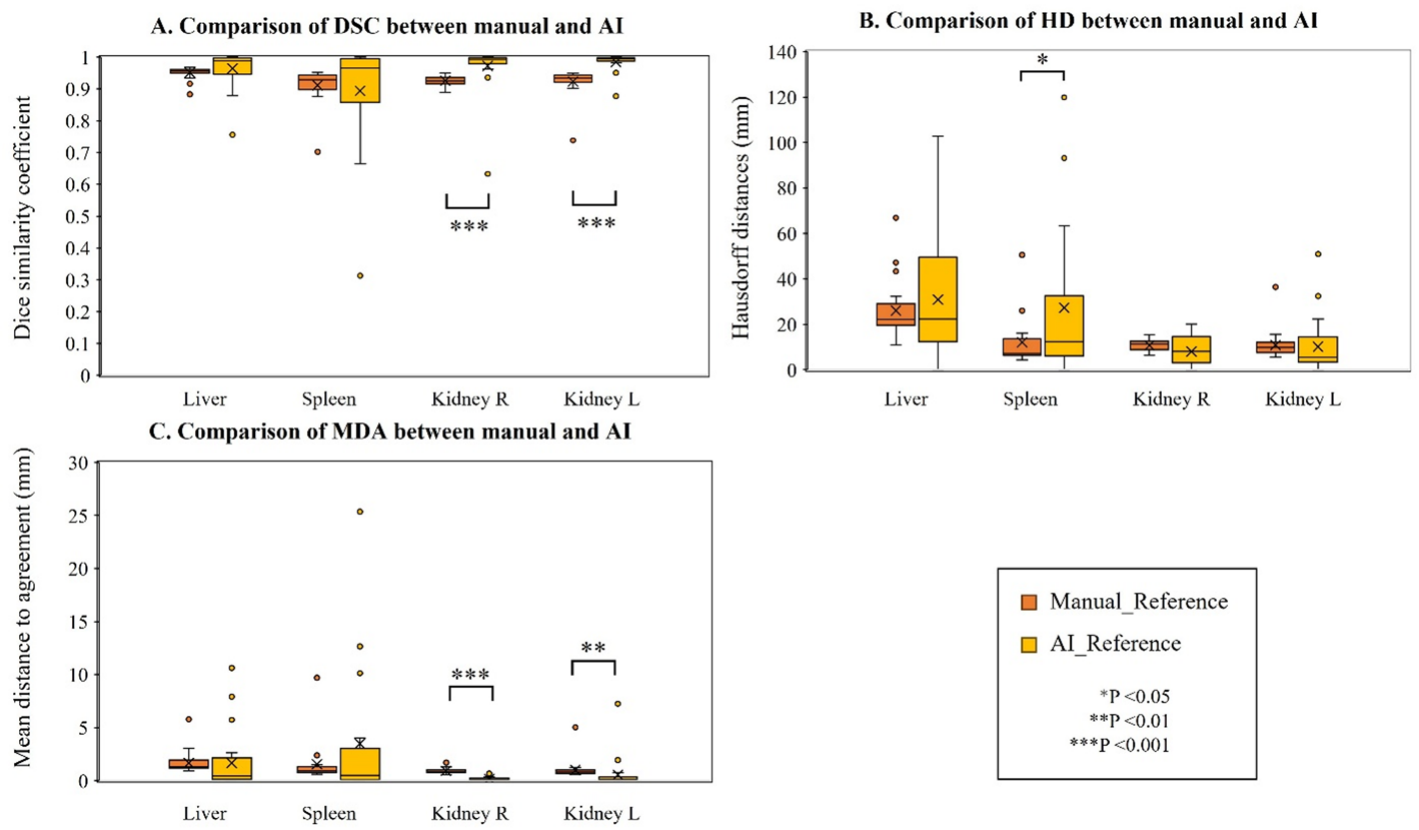
The DSC (**A**), HD (**B**), and MDA (**C**) between manual contouring and AI segmentation and the reference contour in organs, including whole liver (with lesions), spleen, and right and left kidneys, between manual contouring and AI segmentation and the reference contour. The symbol of “*” indicates a statistically significant difference. DSC; Dice similarity coefficients, HD; Hausdorff distances, MDA; mean distance to agreement, AI; artificial intelligence

Figure 2 and Table 2 present comparisons of DSC, HD, and MDA between manual contouring and AI segmentation using the reference contour of normal organs, including the whole liver, spleen, and left and right kidneys. AI segmentation was significantly superior to manual contouring for DSC and MDA in the right kidney (both *p* < 0.001) and the left kidney (*p* < 0.0001 and *p* = 0.001, respectively). For HD in the spleen, manual contouring showed significantly lower values than those of AI segmentation (*p* = 0.027). In evaluating contour accuracy, the median DSC and MDA in all normal organs satisfied the tolerance (≥ 0.800 and ≤ 3.0 mm, respectively) for manual contouring and AI segmentation. However, the median HD for all normal organs exceeded the tolerance regardless of the contouring method. The proportion of cases meeting the criteria for the contouring accuracy metrics, including DSC, HD, and MDA, was compared between manual contouring and AI, using the reference contouring in each OAR. In contrast to HD, DSC and MDA met the criteria in most cases. Cases in which AI segmentation showed outlier liver (3/23) and spleen (2/23) HD (HD > 30.0 mm) were due to hepatomegaly caused by metastatic whole liver tumors.

### Dosimetry

**Fig. 3:**
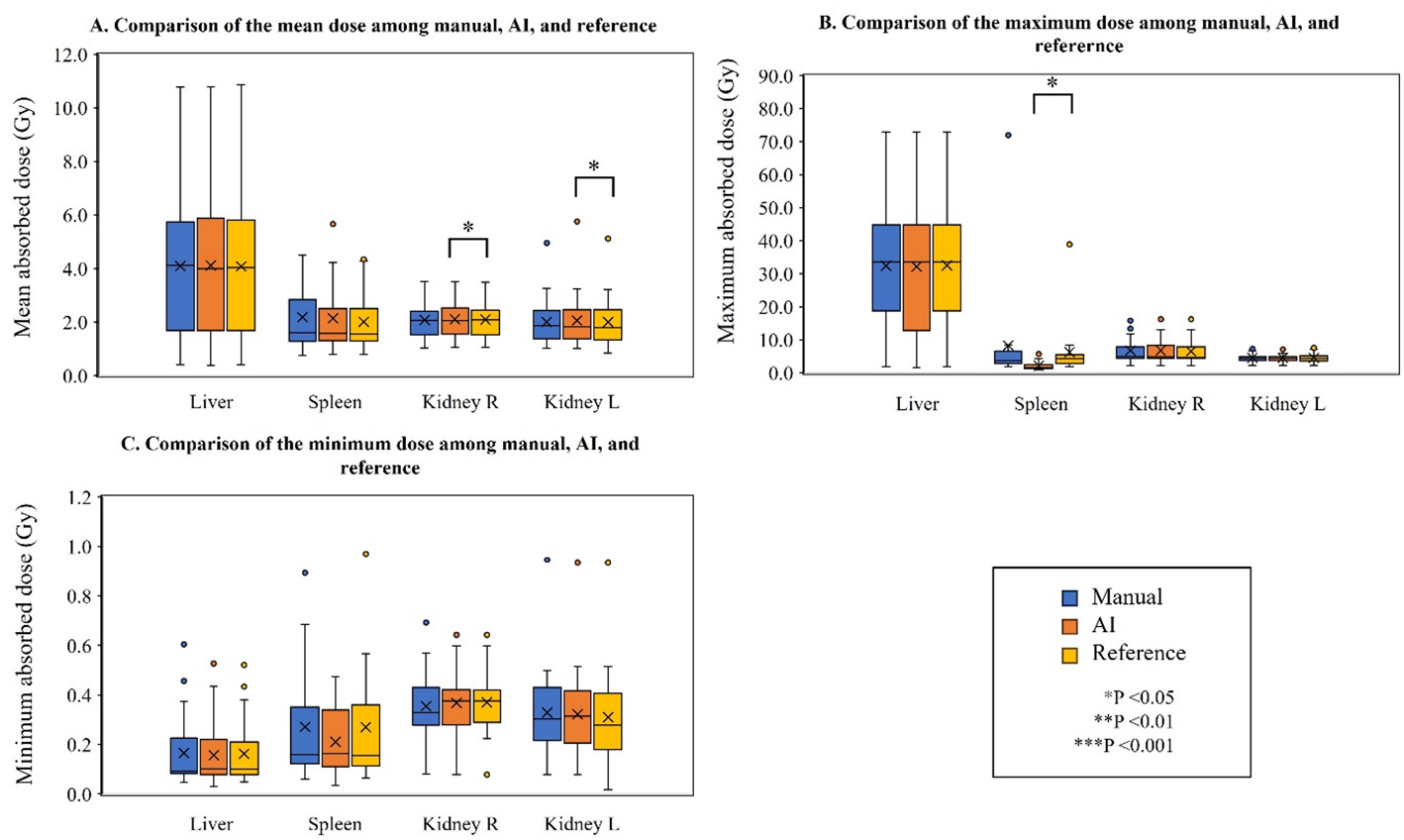
The D_mean_ (**A**), D_max_ (**B**), and D_min_ (**C**) of organs, including the whole liver (with lesions), spleen, and right and left kidneys, for manual contouring, AI segmentation, and the reference contour. The symbol of “*” indicates a statistically significant difference. D_mean_; mean absorbed dose, D_max_; maximum absorbed dose, D_min_; minimum absorbed dose, AI; artificial intelligence

Figure 3 and Table 3 show comparisons of D_mean_, D_max_, and D_min_ for manual contouring, the AI segmentation, and the reference contour for all normal organs, including the whole liver (lesions), spleen, and left and right kidneys. AI segmentation provided significantly higher D_mean_ values than those of the reference contour for the right and left kidneys (*p* = 0.017 and 0.043, respectively). The median relative difference between AI segmentation and the reference contour was 0.5% for the right kidney and 2.2% for the left kidney. The D_max_ for the spleen in AI segmentation was significantly lower than that in the reference contours (*p* < 0.001).

### Time required

**Fig. 4:**
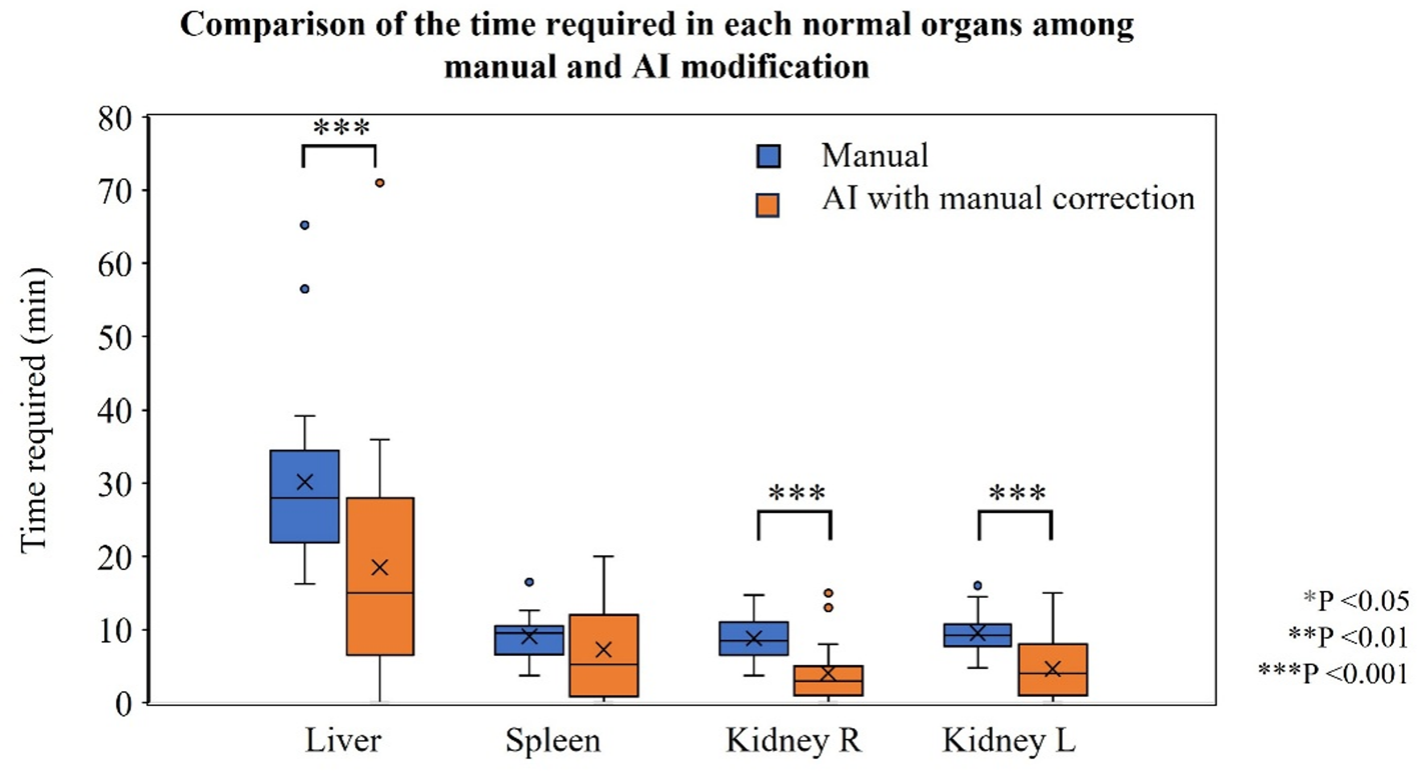
The time required to delineate each organ, including whole liver (with lesions), spleen, and right and left kidneys, in manual contour and AI segmentation with manual corrections. AI segmentation with correction was statistically significantly faster than manual contouring except for the spleen. The symbol of “*” indicates a statistically significant difference. AI; artificial intelligence

The time required for SPECT reconstruction, absorbed dose calculation, and AI segmentation was 1.5 (1.5–1.5) min, 1.5 (1.5–1.5) min, and 11.0 (11.0–15.0) min, respectively. Figure 4 and Table 4 present the time required for manual contouring and the reference contour created with the corrections after AI segmentation in each normal organ. The time required for the reference contour in the whole liver (including lesions), right kidney, and left kidney was significantly shorter than that for manual contouring. The total required time for manual contouring and the reference contour was 54.3 (49.5–67.0) min and 47.0 (30.0–58.0) min, respectively. Further, the total required time for the reference contour, including the corrections after AI segmentation, was significantly shorter than that for manual contouring (*p* = 0.014).

## Discussion

This study investigated the effectiveness of AI segmentation in ^177^Lu-DOTATATE dosimetry automation process by analyzing the accuracy of AI segmentation in OARs and examining the impact on dosimetry and the time required for the tasks, including image reconstruction, contouring, and absorbed dose calculation. Furthermore, we analyzed the accuracy of manual contouring by medical physicists and radiological technologists with different experiences to investigate the potential for reducing the workload on physicians.

Our results demonstrated that the accuracy of AI segmentation measured by the DSC in the normal organs, including the whole liver, spleen, and kidneys, satisfied the tolerance threshold (DSC ≥ 0.800). The AI segmentation of the kidneys demonstrated high spatial concordance with the reference contour; however, minor discrepancies in absorbed dose were noted, attributed to variations in the representation of the renal pelvis and blood vessels. In assessing the contouring accuracy of the whole liver with AI segmentation, HD, which represented a localized segmentation error, exhibited outliers in certain cases because of hepatic enlargement caused by advancing NETs. These errors may impact on the precision of contouring and the absorbed dose in the spleen adjacent to the enlarged liver. The time required for the dosimetry process (SPECT reconstruction, contouring, and absorbed dose calculation) in clinical scenarios was faster when manual correction was added to AI segmentation than when manual contouring was used for all normal organs. We emphasize that AI segmentation may be useful for time efficiency even when contour correction of the liver or spleen is required due to hepatomegaly. Furthermore, this study demonstrated that manual contouring by medical physicists and radiological technologists achieved sufficient accuracy (median DSC ≥ 0.926) without affecting absorbed dose calculation accuracy. Therefore, they have the potential to replace physicians in contouring (correction) tasks for normal organs (except for the liver, including lesions). Nevertheless, since high-dimensional dose assessment metrics such as the DVH indices have not been evaluated, the impact of contouring accuracy on such dosimetric indices should be carefully considered.

The dosimetry of relevant normal organs in ^177^Lu-DOTATATE is essential for accurately predicting and assessing adverse events [25], considering the patient’s radiation treatment history, and facilitating future treatment decisions. Thus, we performed dosimetry on the whole liver (including lesions) and spleen in addition to the kidneys. In the DSC evaluation, all OARs using AI segmentation met the tolerance threshold (≥ 0.800). Specifically, the DSC of kidneys in the AI segmentation showed significantly higher values than those in manual contouring performed with the medical physicists and radiological technologists (right kidney; 0.993, left kidney; 0.994). The D_mean_ of the kidneys derived from the AI segmentation was significantly higher than that from the reference contour. The median difference reached approximately 1.4% (0.5% for the right kidney and 2.2% for the left kidney). In previous reports, spatial concordance indices, including DSC and Jaccard similarity coefficients, have generally reported acceptable values for kidneys [9–11]. Dewaraja et al. reported a DSC of 0.91 between the AI-segmented and reference contours of kidneys, with an absolute dose difference of 2.5% using the minor convolutional neural network (CNN) model in SurePlan™ MRT [9]. A slight difference was observed between their findings and those of this study, which may be attributed to discrepancies in CT images among the enrolled patients, potentially affecting the accuracy of AI segmentation and the variability of the reference contours. However, it can be suggested that the inter-operator variability of the reference contour does not affect the mean absorbed dose of the kidneys because the absolute dose differences were comparable to those of a previous report [9]. Furthermore, the absorbed dose differences for kidneys in our AI segmentation model appear to depend on the learning model in the renal pelvis and vascular delineation. Conversely, another study using a Mask-RCNN model to reduce computational costs reported that the absorbed dose difference may depend on patient anatomy rather than the segmentation model [10]. Notably, absorbed dose characteristics may differ between AI models.

DSC can evaluate the percentage of spatial volume agreement between two structures but cannot adequately evaluate the quantitative relationship between contours or local contour errors [23]. In our study, DSC and MDA met the tolerance threshold in the liver and spleen, but HD was approximately 20.0 mm. This result was mainly due to liver tumors and hepatomegaly, which obscured the boundaries of the spleen, intestinal tract, and blood vessels. The results showed a statistically significant difference in D_max_ of the spleen by AI segmentation. Spleen absorbed doses are also used to assess hematologic toxicity; hence, accuracy has to be checked carefully when the spleen contour from the AI segmentation for dosimetry is used [26]. Additionally, defining the spleen clearly in some cases with non-contrast CT images is difficult; therefore, it may be necessary to consider using other modalities, such as contrast-enhanced CT or magnetic resonance imaging. Furthermore, few reports exist on the segmentation accuracy of the liver and spleen in patients with advanced NET. A previous study utilized AI segmentation for the whole liver, but its accuracy was only visually assessed [9]. Our study results align with those of another study evaluating the whole liver segmentation accuracy using DSC [10]. In this study, we quantitatively evaluated the insufficient segmentation accuracy of the whole liver (including lesions) in patients with advanced NET using spatial concordance indices such as HD and MDA. When the segmentation of the entire liver is insufficient, differences in DVH evaluation may occur. In the patient population of this study, extensive infiltrative tumors were observed throughout the liver in many cases, making tumor segmentation using non-contrast CT difficult. Therefore, when evaluating the segmentation accuracy of the whole liver, including the tumor area, no statistically significant difference was observed in the average liver absorbed dose. We emphasize that liver absorbed dose measurement is essential for determining the combination of dose distributions between past treatments and future treatment plans. Duan et al. investigated adverse events in the kidney and liver during PRRT and found that all patients who developed grade 2 hepatotoxicity had a history of transarterial chemoembolization (TACE), suggesting an association with TACE-induced hepatocyte apoptosis and perivascular fibrosis [27]. Similarly, it may be necessary to carefully investigate the relationship between a history of external radiation therapy and absorbed dose in PRRT.

For absorbed dose assessments, a time-integrated activity curve was calculated using the Hänscheid et al. method, which is a single time point method installed in MIM SurePlan, owing to resource constraints with the SPECT/CT systems. Although this method ensures accuracy of absorbed dose calculation from 72 to 96 h after administration in ^177^Lu-DOTATATE, the median time from the time of administration to the start of imaging was 24.49 h at our facility because SPECT/CT images were taken immediately after discharging the patient. Therefore, in this study, the absorbed doses for the kidneys, whole liver (including lesions), and spleen calculated using the Hänscheid approach might be underestimated by approximately 31.1%, 43.3%, and 43.9%, respectively. The mean absorbed dose for kidneys in our study was underestimated by 32.7% compared to that reported by Dewaraja et al. and was consistent with a previous study [9, 20]. We performed the relative evaluation of organ absorbed doses among different contouring methods; hence, the effect of absorbed dose errors depending on the estimation of the time-integrated activity curve method might be minimal. Furthermore, a recent study has explored improving absorbed dose calculation accuracy using a single time point, addressing challenges similar to those in this study [28]. Dosimetry may also be essential in determining individual dosing regimens, as a study indicates that the total tumor absorbed dose is related to treatment efficacy [29]. The results of this study highlighted the importance of the dosimetry process and the problems associated with its current widespread use in nuclear medicine therapy.

The essential significance of introducing AI segmentation lies in improving time efficiency and reducing mental and physical burdens. Therefore, it is necessary to compare the time required for individual organs and examine the effectiveness of implementing AI segmentation under more realistic clinical scenarios. We examined this effectiveness by comparing the time required for all processes in the ^177^Lu-DOTATATE dosimetry, including contouring, image reconstruction, and absorbed dose calculation, using a commercially available dose calculation engine. Consequently, comparing the time required for delineating normal organs, excluding the spleen, showed that AI segmentation with certain manual corrections significantly reduced the time required compared to manual contour delineation. The correction for a kidney took less than 4 min, while that for the whole liver (including lesions) and spleen took 15.0 and 5.3 min, respectively. In our study, when only the kidneys were subjected to dosimetry, the total time required, including the other processes, was 24.0 (18.0–29.0) min. A previous study that investigated the time required for each process in a single patient case reported 2 min for CNN segmentation, 3 min for SPECT alignment, and 4 min for 4-time points for Monte Carlo absorbed dose calculation, for a total of 25 min (2 min for manual operation) [9]. The study also reported that AI segmentation with the corrections ranged from 0.5 to 3 min per organ. Despite the influence of CPU processing performance and differences in calculation algorithms, the time required for the entire process was comparable to that in our study. The slight difference in the time required to correct the AI segmentation may be attributed to differences in slice thickness and image acquisition range of CT images. For dosimetry, including the whole liver (including lesions), spleen, and kidneys, AI segmentation with the correction significantly reduced the time required for all processes. However, inter-patient variability in the whole liver segmentation accuracy in advanced NET remained considerable, requiring up to nearly 2 h to correct contours. In this study, a 1.5 mm slice thickness of CT was used for the AI segmentation to accurately determine the anatomical location of SPECT accumulation. Increasing CT slice thickness can further reduce the processing time for the AI segmentation and the manual correction time while paying careful attention to the absorbed dose evaluation. Additionally, AI segmentation can be performed in parallel with other priority tasks, which is expected to reduce the mental and physical burden of the operators. However, the inadequate contouring of the whole liver and spleen is clinically unacceptable. More flexible architectures that are independent of whole liver volume and contrast with surrounding organs are expected to be developed.

The radionuclide therapy, which is expected to spread in the future [30], may impose an additional workload on physicians and radiological technologists. The European Association of Nuclear Medicine Internal Dosimetry Task Force surveyed the nuclear medicine treatment work of medical physicists to ensure compliance with Council Directive (2013/59/Euratom) [31], which calls for their active involvement in the standardization of nuclear medicine treatment [15]. Generally, the delineation of normal organs for dosimetry has been performed by a physician or an experienced medical physicist or radiological technologist (with physician review) [9, 10]. However, the potential involvement of medical physicists and radiological technologists has yet to be adequately discussed. In this study, the accuracy of normal organ contouring by medical physicists and radiological technologists with varying levels of experience was examined and found to have adequate contouring accuracy (DSC > 0.926), although inferior to AI segmentation. Additionally, HD in manual contouring tended to be low compared to AI segmentation, especially for the liver and spleen, indicating considerable local errors in their contouring of normal organs. Moreover, the minimal effect on the mean absorbed dose suggests that corrections to the AI segmentation by medical physicists and radiological technologists may contribute to the efficiency of dosimetry. Consensus was required for contouring procedures, as sufficient accuracy was achieved by pre-defining and sharing rules for the renal pelvis and blood vessels. The delineation of serial organs (organs in which even radiation damage to a small volume can cause dysfunction of the entire organ) is directly related to serious adverse events and should be confirmed by a physician.

This research presents several limitations. The reference contours were initially generated by altering AI segmentation. While the results may potentially exaggerate the contour accuracy of AI segmentation, the accuracy of contour delineation aligns with prior studies, suggesting no substantial impact. Furthermore, this study aimed to examine the duration needed for modifications to assess the efficacy of implementing AI segmentation in clinical scenarios. MIRD Pamphlet 17 outlines the necessity of assessing the robustness of quantification under reconstruction conditions; however, the requisite number of iterations has not been adequately examined. The influence of CT image quality on the precision of AI segmentation remains unexamined. The influence of image quality is contingent upon the AI model; however, CT is typically employed for absorption correction in SPECT imaging, necessitating careful consideration of image quality effects in low-dose CT. Although corrections may be required for contours lacking precision due to image quality, it is crucial to highlight that the implementation of AI segmentation offers benefits in time efficiency.

## Conclusions

We investigated the effectiveness of AI segmentation in ^177^Lu-DOTATATE dosimetry for patients with advanced NETs, focusing on contouring accuracy, dosimetric impact, and time efficiency. The contouring accuracy of the whole liver (including lesion), spleen, and kidneys met the DSC acceptable criteria ($\:\ge\:\:$0.8). Kidney segmentation showing strong agreement with the reference contour (median DSC: 0.994, median MDA: 0.1 mm), but slight differences in kidney absorbed doses (right kidney: 0.5%, left kidney: 2.2%) were observed, likely attributable to variation in vessels and renal pelvis delineation. In the whole liver and spleen, the maximum absorbed dose difference was observed due to local contouring errors (evaluated by HD) caused by hepatomegaly. These findings highlight the need for caution when interpreting higher-dimensional dose metrics, such as DVH indices. Regarding the entire dosimetry process, manual correction of AI segmentation significantly reduced the total required time compared to manual contouring alone. Even in cases requiring substantial correction due to hepatomegaly, the integration of AI segmentation remains advantageous for streamlining clinical operations. Additionally, contours produced by medical physicists and radiological technologists demonstrated sufficient accuracy to ensure reliable average absorbed dose estimation. This suggests that, with appropriate oversight, these staff may effectively support or even replace physician-led normal organ contouring in routine dosimetric workflows.

**Table 1:** Patients summary. Age, weight, and eGFR were expressed as median (minimum – maximum)

Age	60 (25–78)	
*Sex*	14: 9 (male: female)	
*Weight (kg)*	54.7 (40.3–75.2)	
*eGFR (mL/min/1.73 m* ^*2*^ *)*	69 (23–146)	
*Primary tumor sites*	Pancreas	16
	Rectum	2
	Gastrointestinal	2
	Unknown	3

eGFR; estimated glomerular filtration rate

**Table 2:** The contouring accuracy and rate of cases meeting the criteria, including Dice similarity coefficient (DSC), Hausdorff distance (HD), and mean distance-to-agreement (MDA) between manual and artificial intelligence segmentation and reference contour, in organs (liver, spleen, and kidneys)

Contouring accuracy									
		Liver^※1^	*P*-value	Spleen	*P*-value	Right kidney	*P*-value	Left kidney	*P*-value
*DSC*	*Manual*	0.955 (0.950–0.961)	0.105	0.929 (0.898–0.944)	0.517	0.926 (0.916–0.935)	< 0.001***	0.935 (0.921–0.943)	< 0.001***
	*AI*	0.988 (0.946–0.998)		0.965 (0.858–0.995)		0.993 (0.979–0.999)		0.994 (0.987–0.999)	
*HD (mm)*	*Manual*	22.2 (19.6–29.0)	0.665	7.1 (6.3–13.7)	0.027*	11.3 (8.9–12.6)	0.080	9.9 (7.7–12.0)	0.300
	*AI*	22.4 (12.4–49.5)		12.5 (6.1–32.6)		8.2 (3.1–14.6)		5.5 (3.5–14.5)	
*MDA (mm)*	*Manual*	1.3 (1.2–1.9)	0.200	0.9 (0.8–1.3)	1	0.9 (0.8-1.0)	< 0.001***	0.8 (0.7-1.0)	0.001**
	*AI*	0.4 (0.1–2.1)		0.5 (0.1–3.1)		0.1 (0.0-0.2)		0.1 (0.0-0.4)	

Rate of cases meeting the criteria									
		Liver^※1^		Spleen^※2^		Right kidney		Left kidney	
*DSC* ($\:\ge\:0.8)$	*Manual*	100% (23 / 23)		94.1% (16 / 17)		100% (23 / 23)		95.7% (22 / 23)	
	*AI*	95.7% (22 / 23)		88.2% (15 / 17)		95.7% (22 / 23)		100% (23 / 23)	
*HD* ($\:\le\:3\:\mathrm{m}\mathrm{m})$	*Manual*	0% (0 / 23)		0% (0 / 17)		0% (0 / 23)		0% (0 / 23)	
	*AI*	4.3% (1 / 23)		11.8% (2 / 17)		21.7% (5 / 23)		17.4% (4 / 23)	
*MDA* ($\:\le\:3\:\mathrm{m}\mathrm{m})$	*Manual*	91.3% (21 / 23)		94.1% (16 / 17)		100% (23 / 23)		95.7% (22 / 23)	
	*AI*	87.0% (20 / 23)		76.5% (13 / 17)		100% (23 / 23)		95.7% (22 / 23)	

*P < 0.05, **P < 0.01, ***P < 0.001

※1: Liver includes lesions

※2: Six of 23 cases had no spleen

Data were expressed as median (1st quartile-3rd quartile)

**Table 3:** The absorbed dose, including mean dose (D_mean_), maximum dose (D_max_), and minimum dose (D_min_) of manual contour, artificial intelligence (AI) segmentation, and reference contour in organs (liver, spleen, and kidneys)

		Liver^※1^	*P*–value	Spleen	*P*–value	Right kidney	*P*–value	Left kidney	*P*–value
*Dmean (Gy)*	*Manual*	4.11 (1.68–5.75)	0.823	1.60 (1.29–2.84)	0.087	2.07 (1.53–2.41)	0.056	1.86 (1.37–2.44)	0.273
	*AI*	4.00 (1.70–5.89)	1	1.58 (1.31–2.50)	0.754	2.07 (1.56–2.53)	0.017**	1.82 (1.38–2.46)	0.043*
	*Reference*	4.03 (1.69–5.81)	–	1.55 (1.30–2.50)	–	2.06 (1.55–2.43)	–	1.78 (1.36–2.45)	–
*Dmax (Gy)*	*Manual*	33.55 (18.71–44.8)	1	3.65 (2.85–6.50)	0.529	4.93 (4.36–7.81)	0.675	4.36 (3.72–4.94)	0.944
	*AI*	33.55 (12.79–44.77)	0.584	1.58 (1.31–2.50)	< 0.001***	4.76 (4.35–8.27)	0.416	4.36 (3.72–4.94)	1
	*Reference*	33.55 (18.71–44.77)	–	4.19 (2.75–5.49)	–	4.72 (4.35–8.22)	–	4.36 (3.72–4.94)	–
*Dmin (Gy)*	*Manual*	0.09 (0.08–0.23)	0.673	0.16 (0.12–0.35)	0.818	0.33 (0.28–0.43)	0.247	0.30 (0.22–0.43)	0.065
	*AI*	0.10 (0.08–0.22)	0.824	0.16 (0.11–0.34)	0.625	0.38 (0.28–0.42)	0.584	0.31 (0.21–0.42)	0.205
	*Reference*	0.10 (0.08–0.21)	–	0.16 (0.11^0.36)	–	0.38 (0.28–0.42)	–	0.30 (0.18–0.42)	–

*P < 0.05, **P < 0.01, ***P < 0.001

The doses for the manual contours and the AI segmentation were compared to those for the reference contours. Data were expressed as median (1st quartile-3rd quartile)

※1: Liver includes lesions

**Table 4:** The time required for contouring by manual and artificial intelligence segmentation with manual corrections in organs, including the liver, spleen, and kidneys

		Liver^※1^	*P*-value	Spleen	*P*-value	Right kidney	*P*-value	Left kidney	*P*-value
*Time (min)*	*Manual*	28.0 (21.9–34.5)	< 0.001***	9.5 (6.6–10.5)	0.243	8.5 (6.5–11.0)	< 0.001***	9.3 (7.8–10.8)	< 0.001***
	*AI correction*	15.0 (6.5–28.0)		5.3 (0.9–12.0)		3.0 (1.0–5.0)		4.0 (1.0–8.0)	

Data were expressed as median (1st quartile-3rd quartile)

※1: Liver includes lesions

*P < 0.05, **P < 0.01, ***P < 0.001

## Electronic Supplementary Material

Below is the link to the electronic supplementary material.

Supplementary Material 1

Supplementary Material 2

## Data Availability

The datasets generated and/or analyzed during the current study are available from the corresponding author upon reasonable request.

## Abbreviations

AI: Artificial intelligence
DSC: Dice similarity coefficient
DVH: Dose-Volume Histograms
HD: Hausdorff distance
^177^Lu-DOTATATE: ^177^Lutetium-[DOTA^0^, Tyr^3^] octreotate
MIRD: Medical Internal Radiation Dose
MDA: Mean distance to agreement
NET: Neuroendocrine tumors
OARs: Organs at risk
PRRT: Peptide receptor radionuclide therapy
SPECT/CT: Single photon emission computed tomography combined with computed tomography
