# Supplementary material for: Evaluation of segmentation accuracy and the improvement of time effectiveness using deep learning-based segmentation in 177Lu-DOTATATE dosimetry
Source: EJNMMI Phys. 2026 May 24;13:56. doi: 10.1186/s40658-026-00897-x (PMC13221549; doi:10.1186/s40658-026-00897-x)
Supplement: Supplementary file 1 — Supplementary Material 1 [file 40658_2026_897_MOESM1_ESM.doc]

|  | ***Cover Letter***  ***European journal of nuclear medicine and molecular imaging Physics*** |
| --- | --- |

***Mar 26, 2026***

*Prof. Stefaan Vandenberghe*

*Editor-in-chief*

*EJNMMI Physics*

*Ghent University, Ghent, Belgium*

Dear Sir:

Thank you for considering our paper entitled “*Evaluation of segmentation accuracy and the improvement of time effectiveness using deep learning-based segmentation in ^177^Lu-DOTATATE dosimetry*”, for possible candidate of publication in your journal, ***European journal of nuclear medicine and molecular imaging Physics***. Please find an attached revised version of our article. According to reviewer reports, we modified the manuscript and now the authors believe the article became much better than before. We thank you for taking the time to consider this submission and look forward to hearing from you a positive reply.

Sincerely,

*Satoshi Nakamura, PhD*

*Radiation Safety and Quality Assurance Division,*

*National Cancer Center Hospital,*

*5-1-1 Tsukiji, Chuo-ku, Tokyo 104-0045, Japan*

*Phone: +81-3-3542-2511*
