# Supplementary material for: Evaluation of segmentation accuracy and the improvement of time effectiveness using deep learning-based segmentation in 177Lu-DOTATATE dosimetry
Source: EJNMMI Phys. 2026 May 24;13:56. doi: 10.1186/s40658-026-00897-x (PMC13221549; doi:10.1186/s40658-026-00897-x)
Supplement: Supplementary file 2 — Supplementary Material 2 [file 40658_2026_897_MOESM2_ESM.docx]

Dear reviewers:

Manuscript: EJPH-D-24-00242R2

Title: Evaluation of the contouring accuracy and the improvement of time effectiveness in clinical situations using deep learning-based segmentation in 177Lu-DOTATATE dosimetry

We would like to thank the reviewers for your careful review of our manuscript. Your comments were particularly useful and helped us to improve our manuscript. The positive comments about the manuscript are greatly appreciated, and we have made careful corrections in accordance with your constructive and insightful comments, which are addressed below in a point-by-point manner. The revisions to the manuscript are shown in red and underlined for changes made in response to the reviewers’ comments.

Respectfully yours,

Reviewer reports:

Reviewer #1: Thank you for your efforts on this important topic.

**Comment 1**

The following two statements still appear inconsistent and require clarification: 1. "Four medical physicists and four radiological technologists from the nuclear medicine or radiation oncology department manually contoured the CT images." 2. "Ultimately, each patient's organs were segmented by four individuals with different levels of clinical experience." The first sentence implies that eight individuals performed manual contouring, whereas the second indicates that each patient was segmented by four individuals. My understanding is that each patient was contoured by four individuals, one from each of four experience-based groups (with two individuals per group). If this interpretation is correct, it is unclear what is the purpose of sentence 1 that states that "four medical physicists and four radiological technologists manually contoured the CT images." A clearer and more explicit description of the observer assignment would improve reproducibility and avoid confusion.

**Response:**

Thank you for this helpful comment. We agree that the original wording could cause confusion regarding the number of observers involved in manual contouring. We have revised the manuscript to clarify that eight observers in total participated in the study (four medical physicists and four radiological technologists), divided into four experience-based groups with two individuals per group. The manuscript was revised as follows:

#P10, line19

*Manual contouring*: Eight observers from the nuclear medicine or radiation oncology department participated in the manual contouring process, including four medical physicists and four radiological technologists. These observers were divided into four groups according to clinical experience, with two individuals in each group. For each patient, one observer was randomly selected from each experienced-based group to perform organ segmentation. Consequently, each patient's organs were segmented by four individuals with different levels of clinical experience.

**Comment 2**

The Wilcoxon signed-rank test is appropriate for paired, non-normally distributed data and evaluates differences in medians, not means. The manuscript states that nonparametric tests were used due to limited sample size, which makes sense. However, the results are presented as mean ± standard deviation, which is inconsistent with the chosen statistical approach. For example, the statement: "The total required time for manual contouring and the reference contour was 58.3 ± 15.1 min and 49.0 ± 25.2 min, respectively (p = 0.014)." is difficult to interpret, as p value corresponds to difference in medians, not means. The reported means and standard deviations suggest substantial overlap between distributions though. Please consider reporting median and interquartile range, which would be more appropriate and informative given the use of the Wilcoxon test. Same applies for all the data and tables that were evaluated with paired analysis of the median (not mean)

**Response:**

Thank you for this important comment. We agree that, because the Wilcoxon signed-rank test evaluates paired differences in medians rather than means, reporting the data as mean ± standard deviation was not fully consistent with the statistical approach used. Accordingly, we have revised the manuscript to present the relevant paired data as median and interquartile range (IQR) throughout the text and tables, which we believe provides a more appropriate and informative summary of the nonparametric analyses. The manuscript was revised as follows:

**Abstract section**

Page3, line16

*Results*: Only one liver delineation by an MP required correction. Median (IQR) DSCs for liver, spleen, and kidneys were 0.955 (0.950-0.961), 0.929 (0.898-0.944), and 0.929 (0.918-0.942) for manual, 0.988 (0.946-0.998), 0.965 (0.858-0.995), and 0.994 (0.985-0.999) for AI-seg, respectively. All organs met the acceptable DSC threshold ($\geq$0.8). Median HD in liver exceeded 10.0 mm for both methods, with AI-seg exceeding 30.0 mm in a few cases (liver: 3/23; spleen: 2/23) due to hepatomegaly. The median MDA in manual and AI-seg was below 2.0 mm. Median mean absorbed doses in the reference was 4.03 (1.69-5.81) Gy for liver, 1.55 (1.30-2.50) Gy for spleen, and 2.03 (1.45-2.44) Gy for kidneys. Only the kidneys absorbed dose from AI-seg differed significantly, showing a 1.4% increase. Dosimetry using the reference method took significantly less time than the manual approach (47.0 [30.0-58.0] min vs. 54.3 [49.5-67.0] min, p=0.014).

**Materials and Methods section**

Page13, line23

Data are presented as median (IQR, 1^st^ quartile-3^rd^ quartile).

**Results section**

Page15, line16

In evaluating contour accuracy, the median DSC and MDA in all normal organs satisfied the tolerance (≥ 0.800 and ≤ 3.0 mm, respectively) for manual contouring and AI segmentation. However, the median HD for all normal organs exceeded the tolerance regardless of the contouring method.

Page14, line6

The median relative difference between AI segmentation and the reference contour was 0.5% for the right kidney and 2.2% for the left kidney.

Page15, line11

The time required for SPECT reconstruction, absorbed dose calculation, and AI segmentation was 1.5 (1.5-1.5) min, 1.5 (1.5-1.5) min, and 11.0 (11.0-15.0) min, respectively.

Page15, line17

The total required time for manual contouring and the reference contour was 54.3 (49.5-67.0) min and 47.0 (30.0-58.0) min, respectively.

**Discussion section**

Page16, line23

Furthermore, this study demonstrated that manual contouring by medical physicists and radiological technologists achieved sufficient accuracy (median DSC ≥ 0.926) without affecting absorbed dose calculation accuracy.

Page17, line11

Specifically, the DSC of kidneys in the AI segmentation showed significantly higher values than those in manual contouring performed with the medical physicists and radiological technologists (right kidney; 0.993, left kidney; 0.994). The D_mean_ of the kidneys derived from the AI segmentation was significantly higher than that from the reference contour. The median difference reached approximately 1.4% (0.5% for the right kidney and 2.2% for the left kidney).

Page18, line10

In our study, DSC and MDA met the tolerance threshold in the liver and spleen, but HD was approximately 20.0 mm.

Page21, line20

The correction for a kidney took less than 4 min, while that for the whole liver (including lesions) and spleen took 15.0 and 5.3 min, respectively. In our study, when only the kidneys were subjected to dosimetry, the total time required, including the other processes, was 24.0 (18.0-29.0) min.

Page22, line6

In this study, the accuracy of normal organ contouring by medical physicists and radiological technologists with varying levels of experience was examined and found to have adequate contouring accuracy (DSC > 0.926), although inferior to AI segmentation.

**Comment 3**

In limitation section (page 23) authors introduce abbreviations that were already introduced in introduction "however, computed tomography (CT) is typically employed for absorption correction in single-photon emission computed tomography (SPECT) imaging, necessitating careful consideration of image quality effects in low-dose CT."

**Conclusion section**

Page24, line5

Kidney segmentation showing strong agreement with the reference contour (median DSC: 0.994, median MDA: 0.1 mm), but slight differences in kidney absorbed doses (right kidney: 0.5%, left kidney: 2.2%) were observed, likely attributable to variation in vessels and renal pelvis delineation.

**Response:**

Thank you for valuable comment. We have verified that the abbreviation is already defined and have made the necessary corrections.

#p23, line4

however, CT is typically employed for absorption correction in SPECT imaging, necessitating careful consideration of image quality effects in low-dose CT.
